# Supplementary figures and images for: Transcriptome-wide investigation of stop codon readthrough in Saccharomyces cerevisiae
Source: PLoS Genet. 2021 Apr 20;17(4):e1009538. doi: 10.1371/journal.pgen.1009538 (PMC8087045; doi:10.1371/journal.pgen.1009538)

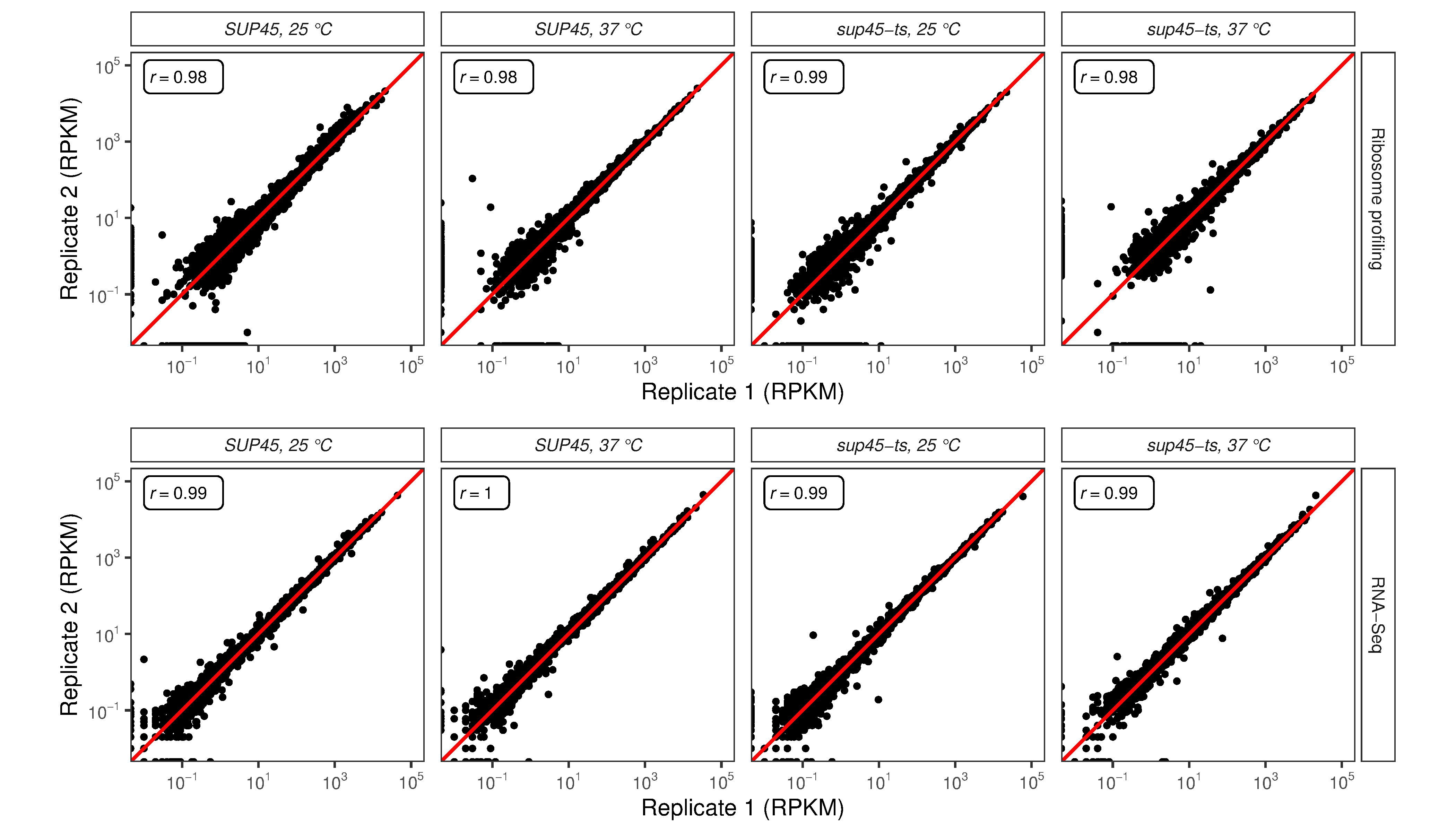

Supplement: S1 Fig — Correlation between ribosome density (top) or mRNA abundance (bottom) in reads per kilobase per million (RPKM) of a pair of replicates for each yeast strain and growth temperature. Pearson’s correlation coefficient (r) was calculated and reported for each pair of replicates. (TIF) [file pgen.1009538.s001.tif]

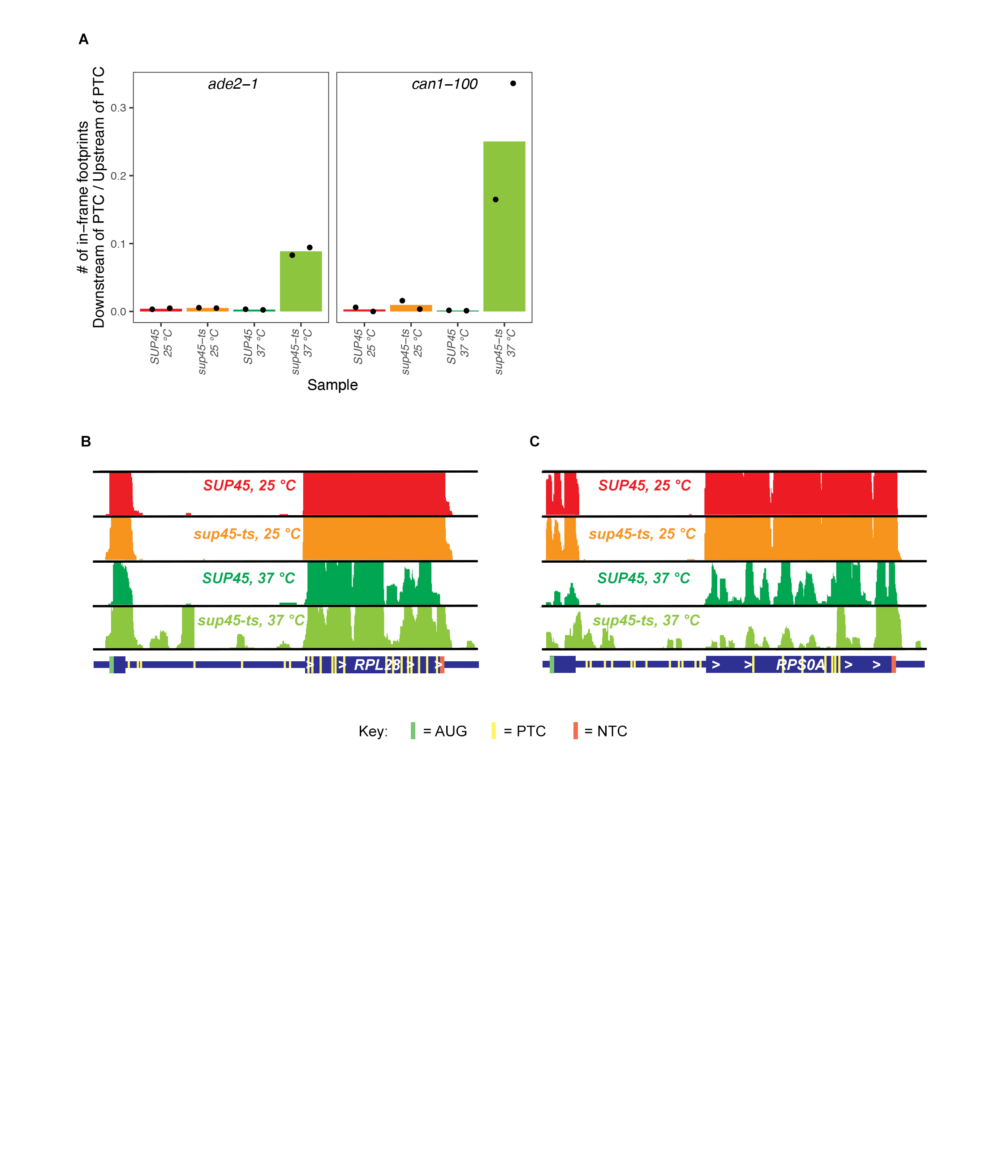

Supplement: S2 Fig — A. Average readthrough efficiency of PTC-readthrough in the ade2-1 and can1-100 alleles. PTC readthrough efficiency was calculated by dividing frame 0 footprint count downstream of the PTC to that upstream of the PTC. Average ± standard deviation of two replicates were plotted for each sample, with black dots indicating the individual data points. B. and C. Read coverage tracks from the Integrative Genomics Viewer (IGV) [69] showing coverage of ribosome profiling reads for the intron-containing genes, B. RPL28 and C. RPS0A, in SUP45 and sup45-ts strains at 25°C and 37°C. Yellow rectangles indicate the position of termination codons in frame with the respective initiation codons under conditions where the introns are translated. Full scale for A and B equals 50 reads. (TIF) [file pgen.1009538.s002.tif]

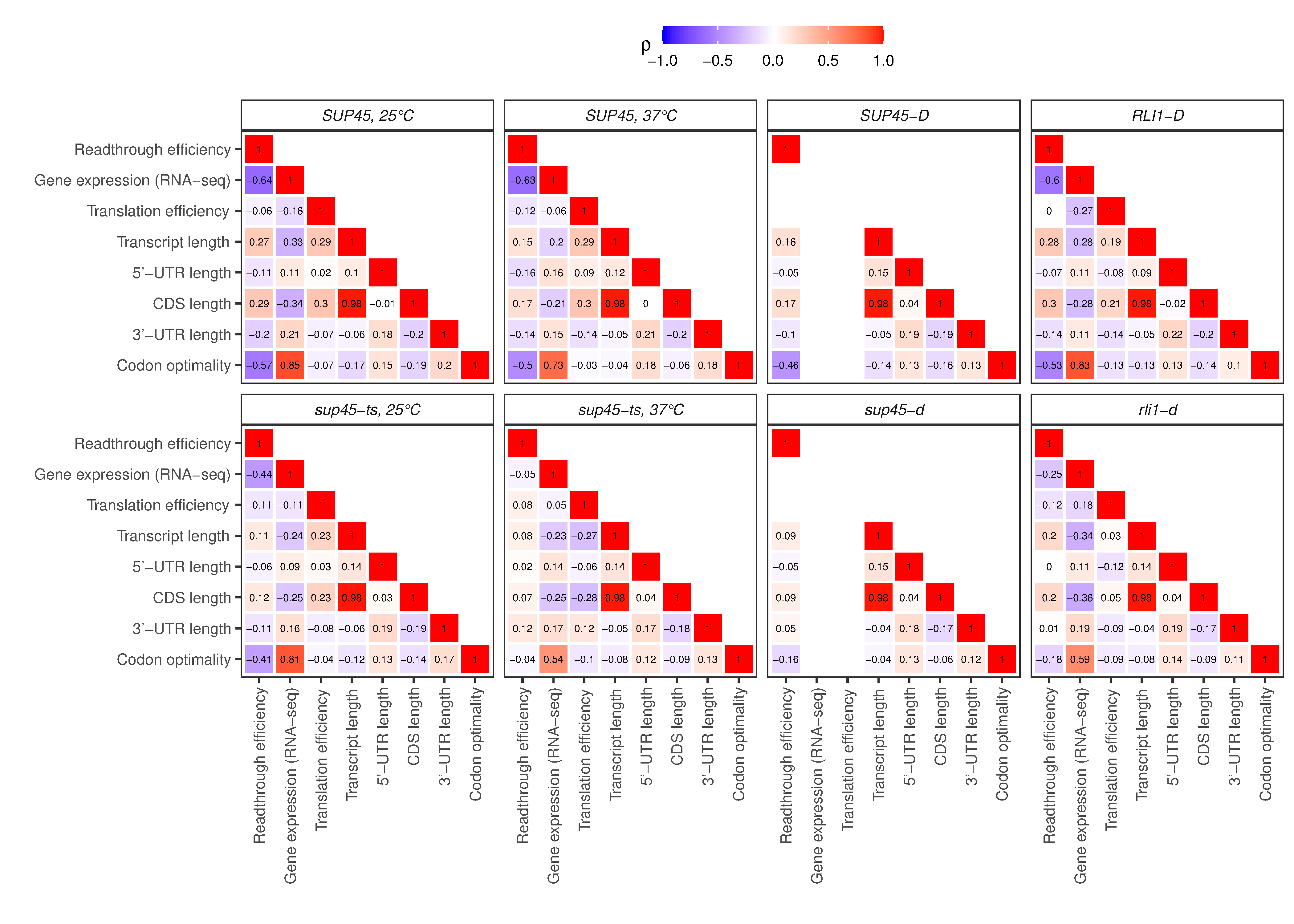

Supplement: S3 Fig — Spearman’s correlation coefficient (ρ) was calculated and reported for each pair of variables. (TIF) [file pgen.1009538.s003.tif]
