# Supplementary material for: Transcriptome-wide investigation of stop codon readthrough in Saccharomyces cerevisiae
Source: PLoS Genet. 2021 Apr 20;17(4):e1009538. doi: 10.1371/journal.pgen.1009538 (PMC8087045; doi:10.1371/journal.pgen.1009538)
Supplement: S1 Table — (PDF) [file pgen.1009538.s004.pdf]

S1 Table. Ribosome profiling data and yeast strains.

Sample	SRR Run	Yeast strain	Notation in this paper	Genotype	Culture media	CHX in lysis buffer
eRF1 temperature-sensitive mutant (This study): GSE162780						
GSM4959785, GSM4959786, GSM4959787, GSM4959788	SRR13208091, SRR13208092, SRR13208093, SRR13208094	WT (HFY114)	*SUP45*	*MATa ade2-1 his3-11,15 leu2-3,112 trp1-1 ura3-1 can1-100*	YPD	No
GSM4959789, GSM4959790, GSM4959791, GSM4959792	SRR13208095, SRR13208096, SRR13208097, SRR13208098	eRF1 ts mutant (HFY1218)	*sup45-ts*	*MATa ade2-1 his3-11,15 leu2-3,112 trp1-1 ura3-1 can1-100 sup45-2*	YPD	No
eRF1 depletion (Wu et al., 2019): GSE115162						
GSM3168380, GSM3168381	SRR7241903, SRR7241904	WT (yCW30)	*SUP45-D*	*MATa his3Δ1 leu2Δ0 met15Δ0 ura3Δ0 HO::ADH1p-OsTIR1-URA3*	YPGR → YPD + 0.5mM auxin	Yes
GSM3168385, GSM3168386	SRR7241908, SRR7241909	eRF1 depleted (yKW13)	*sup45-d*	*MATa his3Δ1 leu2Δ0 met15Δ0 ura3Δ0 KanMX4:*P_{GAL}*1-SUP45*	YPGR → YPD	Yes
Rli1 depletion (Young et al., 2015): GSE69414						
GSM1700885	SRR2046309, SRR2046310	WT (BY4741)	*RLI1-D*	*MATa his3Δ1 leu2Δ0 met15Δ0 ura3Δ0*	YPG → YPD	Yes
GSM1700886, GSM1700891	SRR2046311, SRR2046312, SRR2046319	Rli1 depleted (YDH369)	*rli1-d*	*MATα his3Δ1 leu2Δ0 met15Δ0 ura3Δ0 lys2Δ0 rli1Δ::kanMX4* pDH181 (P_{GAL}*-UBI-R-FH-RLI1, LEU2*)	YPG → YPD	Yes
