# Supplementary material for: Transcriptome-wide investigation of stop codon readthrough in Saccharomyces cerevisiae
Source: PLoS Genet. 2021 Apr 20;17(4):e1009538. doi: 10.1371/journal.pgen.1009538 (PMC8087045; doi:10.1371/journal.pgen.1009538)
Supplement: S2 Table — (PDF) [file pgen.1009538.s005.pdf]

S2 Table. Number of genes involved in statistical analyses.

Sample	Regression	Classification		
		High	Low	Total
SUP45, 25°C	829	125	125	250
SUP45, 37°C	565	85	85	170
sup45-ts, 25°C	1347	202	202	404
sup45-ts, 37°C	2484	373	373	746
SUP45-D	975	147	147	294
sup45-d	2146	322	322	644
RLI1-D	937	141	141	282
rli1-d	2007	301	301	602
