# Supplementary material for: Transcriptome-wide investigation of stop codon readthrough in Saccharomyces cerevisiae
Source: PLoS Genet. 2021 Apr 20;17(4):e1009538. doi: 10.1371/journal.pgen.1009538 (PMC8087045; doi:10.1371/journal.pgen.1009538)
Supplement: S3 Table — (PDF) [file pgen.1009538.s006.pdf]

S3 Table. Ribosome profiling reads processing and alignment statistics.

Sample	SRR Run	Total reads processed	Reads ≥ 15 bp & contained adapters	Reads not aligned to ncRNAs*	Duplicate reads	Reads aligned to transcriptome
SUP45, 25°C (replicate 1)	SRR13208091	67,532,129	57,853,137	25,787,398	3,441,725	20,407,888
SUP45, 25°C (replicate 2)	SRR13208092	61,556,833	51,451,140	18,867,321	7,016,422	10,101,506
SUP45, 37°C (replicate 1)	SRR13208093	49,805,337	44,462,117	14,274,514	1,866,021	11,195,529
SUP45, 37°C (replicate 2)	SRR13208094	64,613,280	55,971,740	24,346,626	5,660,607	16,685,974
sup45-ts, 25°C (replicate 1)	SRR13208095	91,931,471	70,972,671	34,574,953	5,747,485	24,437,810
sup45-ts, 25°C (replicate 2)	SRR13208096	60,891,476	49,432,024	25,181,918	4,148,599	18,939,808
sup45-ts, 37°C (replicate 1)	SRR13208097	77,154,540	55,550,470	21,351,921	3,510,551	14,378,321
sup45-ts, 37°C (replicate 2)	SRR13208098	66,416,665	48,944,777	17,330,578	3,701,109	10,930,634
SUP45-D (replicate 1)	SRR7241903	54,157,010	53,979,859	39,261,094	N/A	39,261,094
SUP45-D (replicate 2)	SRR7241904	32,548,315	32,188,657	21,139,968	N/A	21,139,968
sup45-d (replicate 1)	SRR7241908	46,687,648	45,226,887	34,527,078	N/A	34,527,078
sup45-d (replicate 2)	SRR7241909	32,916,353	32,750,862	20,325,474	N/A	20,325,474
RLI1-D (replicate 1)	SRR2046309	52,021,032	51,768,936	17,545,223	N/A	17,545,223
RLI1-D (replicate 2)	SRR2046310	52,026,256	49,283,307	41,880,568	N/A	41,880,568
rli1-d (replicate 1)	SRR2046311	46,386,847	45,694,917	15,064,692	N/A	15,064,692
rli1-d (replicate 2)	SRR2046312	158,583,030	155,589,606	53,259,434	N/A	53,259,434
rli1-d (replicate 3)	SRR2046319	35,129,965	33,633,653	28,658,467	N/A	28,658,467

*ncRNAs refer to non protein-coding RNAs
