# Supplementary material for: Transcriptome-wide investigation of stop codon readthrough in Saccharomyces cerevisiae
Source: PLoS Genet. 2021 Apr 20;17(4):e1009538. doi: 10.1371/journal.pgen.1009538 (PMC8087045; doi:10.1371/journal.pgen.1009538)
Supplement: S4 Table — (PDF) [file pgen.1009538.s007.pdf]

S4 Table. Offset from 5’ and 3’ ends of footprint to the first nucleotide in the P-site for each footprint length in each sample.

Sample	P-site Offset from 5’/3’ ends for each footprint length (nt)									
	20	21	22	23	27	28	29	30	31	32
SUP45, 25°C (replicate 1)	12/7	13/7	13/8	14/8	11/15	12/15	13/15	13/16	13/17	13/18
SUP45, 25°C (replicate 2)	12/7	13/7	13/8	14/8	11/15	12/15	13/15	13/16	13/17	13/18
SUP45, 37°C (replicate 1)	12/7	13/7	13/8	13/9	11/15	12/15	13/15	13/16	13/17	13/18
SUP45, 37°C (replicate 2)	12/7	13/7	13/8	14/8	11/15	12/15	13/15	13/16	14/16	13/18
sup45-ts, 25°C (replicate 1)	12/7	12/8	13/8	14/8	11/15	12/15	13/15	13/16	13/17	13/18
sup45-ts, 25°C (replicate 2)	12/7	13/7	13/8	14/8	11/15	12/15	13/15	13/16	13/17	13/18
sup45-ts, 37°C (replicate 1)	12/7	12/8	13/8	14/8	11/15	12/15	13/15	13/16	13/17	13/18
sup45-ts, 37°C (replicate 2)	12/7	13/7	13/8	14/8	11/15	12/15	13/15	13/16	13/17	13/18
SUP45-D (replicate 1)	13/6	14/6	15/6	16/6	11/15	12/15	13/15	14/15	14/16	14/17
SUP45-D (replicate 2)	13/6	14/6	15/6	16/6	11/15	12/15	13/15	14/15	15/15	16/15
sup45-d (replicate 1)	13/6	14/6	15/6	16/6	11/15	12/15	13/15	14/15	14/16	14/17
sup45-d (replicate 2)	13/6	14/6	15/6	16/6	11/15	12/15	13/15	14/15	15/15	16/15
RLI1-D (replicate 1)	4/15	5/15	6/15	7/15	11/15	12/15	13/15	14/15	14/16	14/17
RLI1-D (replicate 2)	4/15	5/15	6/15	7/15	11/15	12/15	13/15	14/15	15/15	16/15
rli1-d (replicate 1)	4/15	5/15	6/15	7/15	11/15	12/15	13/15	14/15	14/16	14/17
rli1-d (replicate 2)	4/15	5/15	6/15	7/15	11/15	12/15	13/15	14/15	14/16	14/17
rli1-d (replicate 3)	4/15	5/15	6/15	7/15	11/15	12/15	13/15	14/15	15/15	16/15
